# Lipolytic inhibitor G0S2 modulates glioma stem-like cell radiation response

**DOI:** 10.1186/s13046-019-1151-x

**Published:** 2019-04-05

**Authors:** Yinfang Wang, Yanli Hou, Weiwei Zhang, Angel A. Alvarez, Yongrui Bai, Bo Hu, Shi-Yuan Cheng, Kun Yang, Yanxin Li, Haizhong Feng

**Affiliations:** 1 State Key Laboratory of Oncogenes and Related Genes, Renji-Med X Clinical Stem Cell Research Center, Ren Ji Hospital, Shanghai Cancer Institute, School of Medicine, Shanghai Jiao Tong University, Shanghai, 200127 China; 2 Department of Radiotherapy, Ren Ji Hospital, School of Medicine, Shanghai Jiao Tong University, Shanghai, 200127 China; 3 Ken and Ruth Davee Department of Neurology, Lou & Jean Malnati Brain Tumor Institute, The Robert H. Lurie Comprehensive Cancer Center, Northwestern University Feinberg School of Medicine, Chicago, IL 60611 USA; 4 Department of Neurosurgery, The First Affiliated Hospital of Hainan Medical University, Haikou, 570102 Hainan China; 5 Key Laboratory of Pediatric Hematology and Oncology Ministry of Health, Pediatric Translational Medicine Institute, Shanghai Children’s Medical Center, School of Medicine, Shanghai Jiao Tong University, Shanghai, 200127 China

**Keywords:** G0/G1 switch gene 2 (G0S2), Glioma stem cell (GSCs), 53BP1, Radioresistance

## Abstract

**Background:**

Ionizing radiation (IR) therapy is the standard first-line treatment for newly diagnosed patients with glioblastoma (GBM), the most common and malignant primary brain tumor. However, the effects of IR are limited due to the aberrant radioresistance of GBM.

**Methods:**

Transcriptome analysis was performed using RNA-seq in radioresistant patient-derived glioma stem-like cells (GSCs). Survival of glioma patient and mice bearing-brain tumors was analyzed by Kaplan–Meier survival analysis. Lipid droplet and γ-H2AX foci-positive cells were evaluated using immunofluorescence staining.

**Results:**

Lipolytic inhibitor G0/G1 switch gene 2 (G0S2) is upregulated in radioresistant GSCs and elevated in clinical GBM. GBM patients with high G0S2 expression had significantly shorter overall survival compared with those with low expression of G0S2. Using genetic approaches targeting G0S2 in glioma cells and GSCs, we found that knockdown of G0S2 promoted lipid droplet turnover, inhibited GSC radioresistance, and extended survival of xenograft tumor mice with or without IR. In contrast, overexpression of G0S2 promoted glioma cell radiation resistance. Mechanistically, high expression of G0S2 reduced lipid droplet turnover and thereby attenuated E3 ligase RNF168-mediated 53BP1 ubiquitination through activated the mechanistic target of rapamycin (mTOR)-ribosomal S6 kinase (S6K) signaling and increased 53BP1 protein stability in response to IR, leading to enhanced DNA repair and glioma radioresistance.

**Conclusions:**

Our findings uncover a new function for lipolytic inhibitor G0S2 as an important regulator for GSC radioresistance, suggesting G0S2 as a potential therapeutic target for treating gliomas.

**Electronic supplementary material:**

The online version of this article (10.1186/s13046-019-1151-x) contains supplementary material, which is available to authorized users.

## Introduction

Glioblastoma (GBM), a WHO grade IV brain tumor is the most common and malignant primary cancer of the central nervous system with a grim median survival of 14.6 months upon diagnosis [1]. Radiotherapy is the standard first line treatment for newly diagnosed patients with gliomas, but its effectiveness is limited given the tumor’s intrinsic resistance and propensity for recurrence [2, 3]. Although possible mechanisms have been attributed to GBM resistance to radiation treatments [4–6], the molecular mechanisms regulating radiation resistance of GBM are still unclear.

G0/G1 switch gene 2 (G0S2) was initially identified in lymphocytes through pharmaceutical stimulation of the G0 to G1 cell cycle transition [7, 8]. G0S2, a small 12 kDa protein, localizes to the mitochondria [9, 10], endoplasmic reticulum [11], and lipid droplets within the adipocytes [12]. G0S2 has been shown to play various important roles in cellular functions such as cell proliferation [13], apoptosis [10], and oxidative phosphorylation [9] in humans and mice. G0S2 is demonstrated to function as a lipolytic inhibitor in lipid metabolism to regulate lipid droplet turnover [12], and lipid droplets were identified as a signature of GBM and inversely correlated with GBM patient survival [14]. Acumulated data have indicated that G0S2 is also involved in cancer [10, 15–17], including glioma [18]. However, the function of G0S2 in cancers is still largely unknown.

Here, by analyzing gene expression profiles in glioma stem cells (GSCs) treated with fractionated radiation, we found that G0S2 is significantly upregulated in radioresistant GSCs. We firstly examined G0S2 expression in glioma cells and clinical specimens. We then assessed the role of G0S2 in radiation response in glioma stem-like cells (GSCs) and glioma cell lines. Finally, we determined the mechanism by which G0S2 enhances radioresistance in gliomas.

## Materials and methods

### Cell lines

Glioma U87, LN229, T98G, U251 and LN444 cells were from ATCC (Manassas, VA, USA). Patient-derived glioma stem cell (GSC) lines, GSC 84, GSC 157, GSC 1123 and GSC 83 were from Dr. Ichiro Nakano [19]. GSC cells were maintained in DMEM/F12 supplemented with B27 (1:50), heparin (5 mg/ml), basic FGF (20 ng/ml), and EGF (20 ng/ml), and glioma cells were cultured in 10% FBS/DMEM as we previously described [19, 20].

### Antibodies and reagents

The following antibodies were used in this study: an anti-G0S2 (dilution 1:200; Proteintech, IL, USA); an anti-phospho-Histone H2A.X (Ser139) (dilution1:1000; EMD Millipore, Billerica, MA, USA); anti-53BP1 (dilution1:500), anti-RNF168 (dilution1:500), anti-CXCL5 (dilution 1:1000) and anti-Rad51 (dilution 1:1000)(Abcam, Cambridge, MA, USA). The secondary antibodies were from Jackson ImmunoResearch Laboratories (West Grove, PA, USA). Cell culture media and other reagents were from Invitrogen (Carlsbad, CA, USA), Sigma-Aldrich (St. Louis, MO, USA) or Peprotech (Rocky Hill, NJ, USA).

### Patient samples

Four fresh samples of human GBM samples and paired normal brain (peritumoral) tissues were obtained from Renji Hospital, Shanghai, China.

### Plasmids

G0S2 and RNF168 cDNAs were amplified by RT-PCR from normal human brain tissues and sequenced. The cDNAs were then subcloned into a lentivirus LeGO-iG vector. shRNAs for G0S2 and 53BP1 were purchased from Thermo Fisher Scientific (Waltham, MA, USA). pMT107-His-Ub was described in our previous report [20].

### Cell transfections

Cell transfections were performed as we previously described [20].

### RNA-Seq and differentially expressed gene analysis

Total RNA was extracted and purified using the Qiagen RNeasy Mini kit (Valencia, CA, USA) according to the manufacturer’s instructions. The quality of RNA was assessed by bioanalyzer before sequencing. Libraries for poly(A)^+^ RNA were prepared according to the Illumina protocol. Libraries were sequenced on Illumina HI-SEQ 2500 platforms. The criteria of Differentially Expressed Genes detection in this study are false discovery rate (FDR) < 0.01 and a fold change > 2. Gene Expression Omnibus (GEO) accession code: GSE79772.

### Western blotting assay

Western blotting assay was performed as we previously described [20]. Briefly, cells were lysed, and then the lysates were centrifuged. Protein concentrations were determined with a BCA protein assay kit. Equal amounts of cell lysates (a total of 30 μg of protein) were resolved in a 2X SDS lysis buffer and analyzed.

### Cell apoptosis assay

Cell apoptosis assay was performed using BD Annexin V: FITC Apoptosis Dectection Kit (BD Biosciences, San Jose, CA, USA), according to the manufacturer’s instructions. Cells (5 × 10^5^) were washed in PBS and centrifuged at 200 x g for 5 min. The supernatant was aspirated. The cell pellet was incubated with annexin-V-FITC (at 1 mg/ml in Hepes buffer with 1.8 mM CaCl_2_) for 5–10 min at room temperature, and added 1 ml Hepes containing 10 mg/ml propidium iodide. Cells were analysed immediately by flow cytometry.

### RNA extraction and quantitative real-time PCR analysis

Total RNA was extracted from indicated cells using Trizol (Invitrogen, Carlsbad, CA, USA), according to the manufacturer’s instructions. Quantitative Real-Time PCR was performed in triplicate using the QuantiTect SYBR Green PCR Kit (Qiagen, Valencia, CA, USA) on a Rotorgene 6000 series PCR machine (Corbett Research, Valencia, CA, USA). All mRNA quantification data were normalized to *ACTB*, which was used as an internal control. The following G0S2 primer sets were used: 5′-GGCCTGATGGAGACTGTGTG-3′ and 5′-CTTGCTTCTGGAGAGCCTGT-3′.

### BODIPY 493/503 staining of neutral lipid droplets

GSCs were incubated under normal growth conditions with 100 μM of oleic acid (OA) (Sigma-Aldrich, catalog number: O3008) complexed to albumin at a molar ratio of 8:1for 16 h, and then incubate on BODIPY staining solution in the dark for 15 min at 37 °C according to the manufacturer’s instructions.

### Immunofluorescent staining and confocal microscopy

Cells grown on coverslips were permeabilized with 0.3% Triton X-100-PBS for 15 min and blocked with 3% BSA for 60 min. After overnight incubation of primary antibodies at 4 °C, the slides were incubated with Alexa Fluor-labeled secondary antibodies, and then were further stained with Hoechst 33258 for 10 min and evaluated with a LSM710 confocal microscope (Zeiss, Germany).

### Tumorigenesis studies

Athymic (Ncr nu/nu) female mice at an age of 6–8 weeks (SLAC, Shanghai, China) were used. Mice were randomly divided into 5–6 per group. Four thousand GSCs or 1 × 10^6^ human glioma cells were stereotactically implanted into the brain of the animals as we previously described [20, 21]. Mice were euthanized when neuropathological symptoms developed.

### Radiation treatment

Cells or mice were irradiated at indicated doses with an X-RAD 160 irradiation system (Precision X-Ray, Inc., Kentwood, MI, USA). A radiation shield was used to protect animal body.

### Colony formation assay

Clonogenic survival assay was performed as previously described [22, 23]. Briefly, approximately 5000 cells after IR were seeded in a 0.5% Noble Agar top layer with a bottom layer of 0.8% Noble Agar in each of the triplicate wells of a 24-well plate. TMZ was added into the top layer. Medium was added after plating and changed every 3 days thereafter. Colonies were scored after 2–4 weeks using Olympus SZX12 stereomicroscope.

### Statistical analysis

Statistical analyses were performed in a GraphPad Prism version 5.0 for Windows (GraphPad Software Inc., San Diego, CA, USA). Survival analysis was carried out by Kaplan-Meier analysis and was compared with the log-rank tests. Comparison of treatments was analyzed using One-way ANOVA with Newman-Keuls post-test or a paired two-way Student’s *t* test as we previously described [21]. *P* values less than 0.05 were considered significant.

## Results

### G0S2 is upregulated in radioresistant glioma stem cells

To identify novel mediators of radiation resistance in GBM, we treated a patient-derived glioma stem cell (GSC) line (GSC 1123-C) with repeated fractionated radiation to establish a radioresistant GSC line (GSC 1123-R). As shown in Additional file 1: Figure S1A, we subjected GSC 1123-C to four rounds of fractionated radiation of 6 Gy every 4 days (total 24 Gy), generating GSCs with greater radiation resistance GSC 1123-R, a pool of cells. GSC 1123-R cells at passage 10 or less were further analyzed. As shown in Additional file 1: Figure S1B and S1C, annexin V staining for apoptotic cells revealed that only 6.1% ± 0.5% of GSC 1123-R cells underwent apoptosis during the 48 h after a single-6 Gy dose irradiation, compared with a 11.2% ± 0.4% of GSC 1123-C cells. Clonogenic assays showed that the surviving fraction of cells receiving single 4- or 6-Gy IR was significantly higher for GSC 1123-R cells than for GSC 1123-C cells (Additional file 1: Figure S1D and S1E). This observation demonstrates that GSC 1123-R cells are more resistant to radiation when compared with GSC 1123-C cells, and were stable in radiation resistance.

**Fig. 1:**
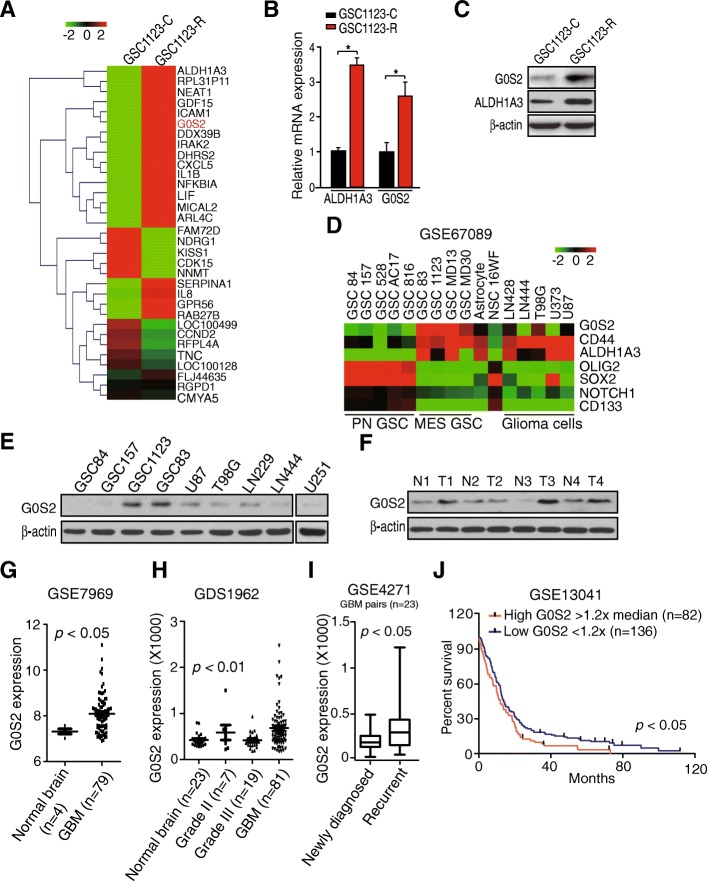
G0S2 is upregulated in radioresistant glioma stem cells (GSCs). **a** Heatmap of mRNA-Seq analysis of differentially expressed genes (2-fold change and FDR < 0.01) between GSC 1123-C and GSC 1123-R cells. **b** Quantitative RT-PCR (QRT-PCR) analysis of *ALDH1A3* and *G0S2* mRNA expression in GSC 1123-C and GSC 1123-R cells. *ACTB* (encoding β-actin) was used as a control. Error bars, SD. *, *p* < 0.05. **c** Western blotting (WB) assays of ALDH1A3 and G0S2 expression in GSC 1123-C and GSC 1123-R cells. **d** Expression level of *G0S2* mRNA in proneural (PN) and mesenchymal (MES) GSCs, neural progenitors (NSC 16WF), normal astrocytes and glioma cell lines from the GSE67089 dataset [19]. **e** WB analysis of G0S2 expression in GSC and glioma cells. β-actin was used as a control. **f** WB analysis of G0S2 expression in four paired clinical GBM samples and normal brain tissues. **g** Expression level of *G0S2* mRNA is significantly higher in GBM compared with normal brains. Expression data of *G0S2* mRNA were downloaded from the GSE7696 dataset [24] and analyzed. **h** Expression level of *G0S2* mRNA is correlated with glioma progression. Expression data of *G0S2* mRNA were downloaded from GSE1962 dataset [25] and analyzed. **i** Expression level of *G0S2* mRNA is higher in recurrent GBM compared with paired newly diagnosed GBM. Expression data of *G0S2* mRNA were downloaded from GSE4271 dataset [44] and analyzed. **j** Kaplan–Meier analysis of patients with high *G0S2* mRNA-expressing glioma tumors versus low *G0S2* mRNA-expressing tumors in GBM from the GSE13041 dataset. Statistical analysis was performed by log-rank test in a GraphPad Prism version 5.0 for Windows. Median survival (in months): low, 12.83; high, 10.58. Black bars, censored data. Data in (B, C, E and F) represent two independent experiments with similar results

Next, we performed transcriptome analysis of GSC 1123-R and GSC 1123-C using RNA-seq. Differential gene expression analysis identified 32 genes that were differentially expressed in GSC 1123-R compared with GSC 1123-C (false discovery rate < 0.01, and a folder change > 2), including *ALDH1A3* (Aldehyde dehydrogenase 1A3) and *G0S2* (Fig. 1a). To validate these RNA-seq results, we performed quantitative real-time PCR (QRT-PCR) analysis of *ALDH1A3* and *G0S2* expression. The data showed an agreement in the expression levels of these genes between the RNA-seq and QRT-PCR analyses (Fig. 1b). We further confirmed that protein expression of G0S2 was higher in GSC 1123-R cells compared with GSC 1123-C cells (Fig. 1c). This result suggests that G0S2 could regulate glioma radioresistance.

We then assessed expression of G0S2 in glioma cells and clinical specimens of patients. We downloaded the GSE67089 dataset [19] and examined *G0S2* mRNA expression in proneural (PN), mesenchymal (MES) subtyped GSCs, astrocytes, 16WF neural stem cells (NSCs) and five established glioma cell lines. As shown in Fig. 1d, *G0S2* was expressed at the highest levels in MES GSCs compared with all other cells. *G0S2* was also co-expressed with MES-associated genes, *CD44* and *ALDH1A3* in MES GSCs [19]. The expression level of G0S2 protein was also the highest in MES GSCs, GSC 1123 and GSC 83 (Fig. 1e) when compared to other cell lines that were analyzed. In clinical tumor samples, compared to paired normal brain tissues, G0S2 was found highly expressed in three of four clinical GBM tissue samples (Fig. 1f). To support our findings, we downloaded GSE7696 [24] and GDS1962 [25] datasets and examined expression level of *G0S2* mRNA in GBM, low grade (WHO grade II and III) and normal brain tissue controls included in these datasets. As shown in Fig. 1g and h, compared with normal brain tissues and low grade gliomas, the expression level of *G0S2* mRNA was significantly elevated in GBM, while no significant differences were measured between normal and low grade gliomas. We also found that the expression level of *G0S2* was markedly higher in recurrent GBM than paired newly diagnosed GBM (Fig. 1i).

Last, we examined the relationship of G0S2 expression and glioma patient survival by Kaplan–Meier survival analysis using the GSE13041 dataset [26]. Segregating patients in the GSE13041 dataset by *G0S2* expression revealed a statistically significant worse prognosis for GBM patients with high *G0S2* (> 1.2 x median level) compared with those with low (< 1.2 x median level) (Fig. 1j). The median patient survival times of these patients were 10.6 and 12.8 months, respectively (*p* < 0.05). This result supports that G0S2 is upregulated in radiation resistant GSCs and may be involved in glioma progression.

### G0S2 mediates lipid droplet turnover and glioma irradiation response

**Fig. 2:**
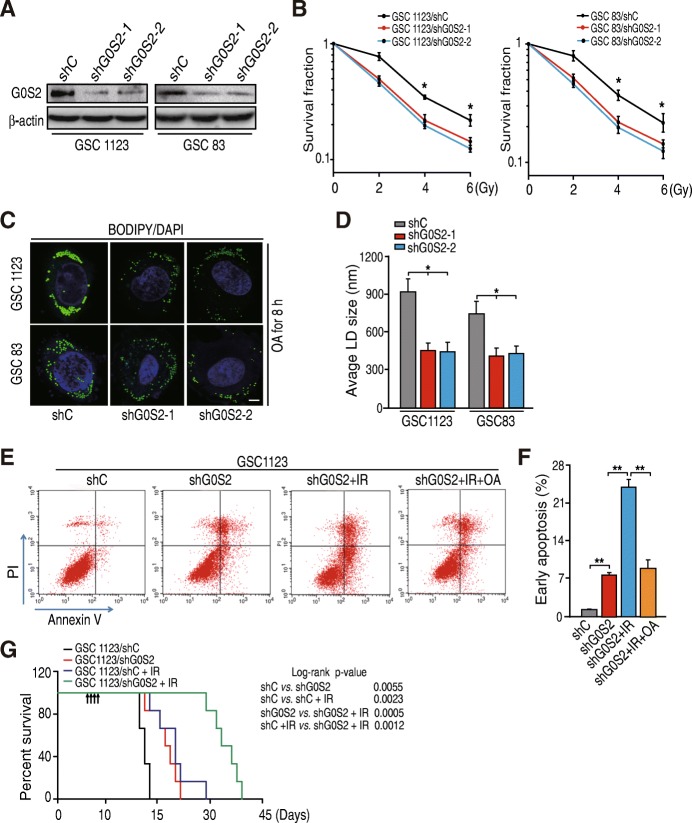
Knockdown of G0S2 enhances glioma radiation response. **a** WB analysis of knockdown of G0S2 with two different shRNAs (shG0S2–1 and shG0S2–2) in GSC 1123 and GSC 83 cell lines. **b** Clonogenic survival assay of GSC 1123 and GSC 83 cell lines transduced with control shRNA (shC) or G0S2 shRNAs (shG0S2–1 and shG0S2–2). Colonies formed by surviving cells 15 days after IR are shown. **c** Immunofluorescence staining with BODIPY 493/503 fluorescence dye was performed to lipid droplets (LD). Two sets of GSCs were incubated under normal growth conditions with 100 μM of oleic acid (OA) complexed to albumin at a molar ratio of 8:1 for 16 h. Bars, 5 μm. **d** Quantification of lipid droplet diameter. An average diameter of 30 lipid droplets per cell over 30 cells for each point was measured. **e** OA treatment inhibited G0S2 shRNA-enhanced cell apoptosis induced by IR. GSC 1123 cells transduced with a shRNA were incubated with 400 μM of OA or vehicle treated with or without 10-Gy IR, and then cell apoptosis were analyzed by FACS. **f** Quantification of early cell apoptosis. **g** Survival curves for mice implantation with 4 × 10^3^ cells of GSC 1123/shC or GSC 1123/shG0S2 and left untreated or given daily dosed 2.5 Gy IR from day 7 to 10 after cell implantation. Arrows, radiation treatment times. Data represent two independent experiments with 6 mice per group with similar results. Error bars, SD. *, *p* < 0.05. **, *p* < 0.01

Since G0S2 is upregulated in radioresistant GSCs, we determined whether G0S2 is involved in glioma radiation resistance. We used lentivirus-mediated short hairpin RNAs (shRNAs) targeting *G0S2* or a non-silencing control to deplete *G0S2* in two patient-derived GSCs, GSC 1123 and GSC 83 (Fig. 2a). We treated GSC 1123/shG0S2, GSC 1123/shC, GSC 83/shG0S2, and GSC 83/shC cells with fractionated irradiation (IR) at clinically relevant doses. As shown in Fig. 2b, knockdown of *G0S2* markedly sensitized GSC 1123 and GSC 83 GSC cells to IR treatments as determined using clonogenic assays.

As G0S2 is a lipolytic inhibitor of adipose triglyceride lipase (ATGL) and regulates lipid droplet turnover [12], we examined whether G0S2-mediated radioresistance is related with G0S2 lipolytic inhibitor activity. GSC 1123 and GSC 83 cells transduced with or without G0S2 shRNAs were incubated in normal medium with oleic acid (OA) for 16 h to promote lipid droplet formation (Fig. 2c). Using BODIPY 493/503, a nonpolar probe selective for neural lipids such as TAG [27], we identified that lipid droplets in GSC 1123/shG0S2 and GSC 83/shG0S2 cells were significantly smaller and lower numerous compared to those in the control cells, respectively (Fig. 2c and d). We then assessed cell apoptosis using fluorescence activated cell sort (FACS). As shown in Fig. 2e and f, knockdown of G0S2 promoted cell apoptosis in GSC 1123 cells, and IR treatment further increased cell apoptosis, whereas OA pre-treatment inhibited IR-induced cell apoptosis in GSC 1123/shG0S2 cells. These results support that G0S2-enhanced radioresistance is related with G0S2-regulated lipid droplet stability.

We further assessed the effect of G0S2 depletion on tumor growth in the brain of mice in response to IR treatment. Ten days after intracranial implantation of GSCs, half of the mice of each group (GSC 1123/shC or GSC 1123/shG0S2) received local IR to the brain for 4 consecutive days at 2.5 Gy/day. Kaplan-Meier survival assay showed that in mice with GSC 1123/shC tumors but without IR treatments, median survival was 17.0 days post-implantation. In contrast, the animals with shG0S2 tumors survived 21.5 days (*p* < 0.01) (Fig. 2g). IR treatment had a modest effect on control animal survival, with median survival times of 17.0 and 23.0 days (*p* < 0.01), respectively. However, the combination of shG0S2 and IR led to the most significant extension of animal survival with a median survival time of 33.0 days when compared to a median survival time of 23.0 days of control mice with IR treatment (*p* < 0.01) (Fig. 2g). This result suggests that a significant benefit to knockdown of G0S2 and radiation over radiation alone.

**Fig. 3:**
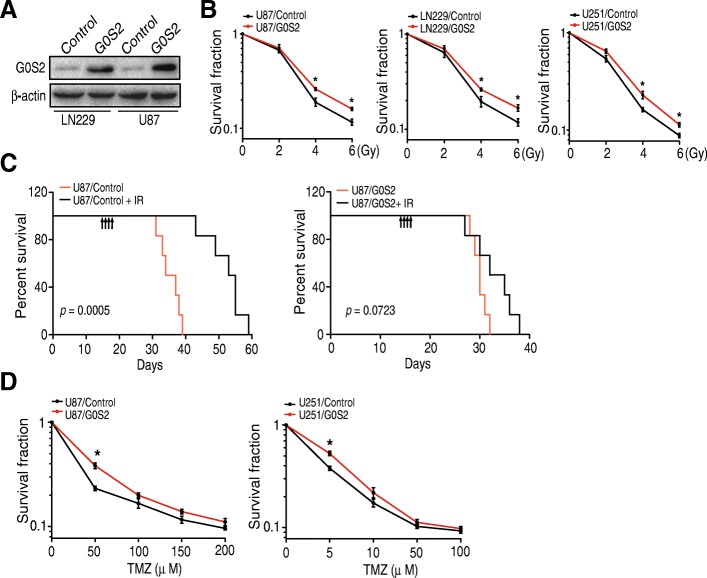
Overexpression of G0S2 reduces glioma radiation response. **a** WB analysis of overexpression of G0S2 in U87, LN229 and U251 cells. **b** Clonogenic survival assay of U87, LN229 and U251 cells transduced with an empty vector (Control) or G0S2. Colonies formed by surviving cells 26 days after IR are shown. Error bars, SD. *, *p* < 0.05. **c**. Survival curves for mice implantation with 5 × 10^5^ cells of U87/Control or U87 /G0S2 and left untreated or given daily dosed 2.5 Gy IR from day 14 to 17 after cell implantation. Arrows, IR treatment times. Data represent two independent experiments with 6 mice per group with similar results. **d** Clonogenic survival assay. Colonies formed by surviving cells 26 days with TMZ treatment are shown. Error bars, SD. *, *p* < 0.05

To further determine the role of G0S2 in glioma response to IR, we stably overexpressed G0S2 in two glioma cell lines with low levels of endogenous G0S2 expression (Fig. 3a), LN229 and U87, and then treated U87/G0S2, U87/Control, LN229/G0S2, LN229/Control, U251/G0S2, and U251/Control with fractionated radiation. As shown in Fig. 3b, overexpression of G0S2 rendered resistance to IR in U87 and LN229 cells compared with the controls, respectively. Consistent with the results in vitro, in mice that received U87 cells, survival of control animals were significantly extended in mice with IR treatments, increasing median survival times from 35.5 to 54.0 days (*p* < 0.001). In contrast, IR treatments did not significantly enhance survival of G0S2-overexpressing glioma tumors (Fig. 3c). The effect of G0S2 overexpression on temozolonide (TMZ) sensitivity was also determined. As shown in Fig. 3d, G0S2-overexpression modestly enhanced TMZ resistance of U87 and U251 GBM cells only in the lower effective doses. This data suggests that G0S2 enhanced survival is greater for radiation responses compared to chemotherapy.

### G0S2 regulates glioma cell DNA repair in response to IR

**Fig. 4:**
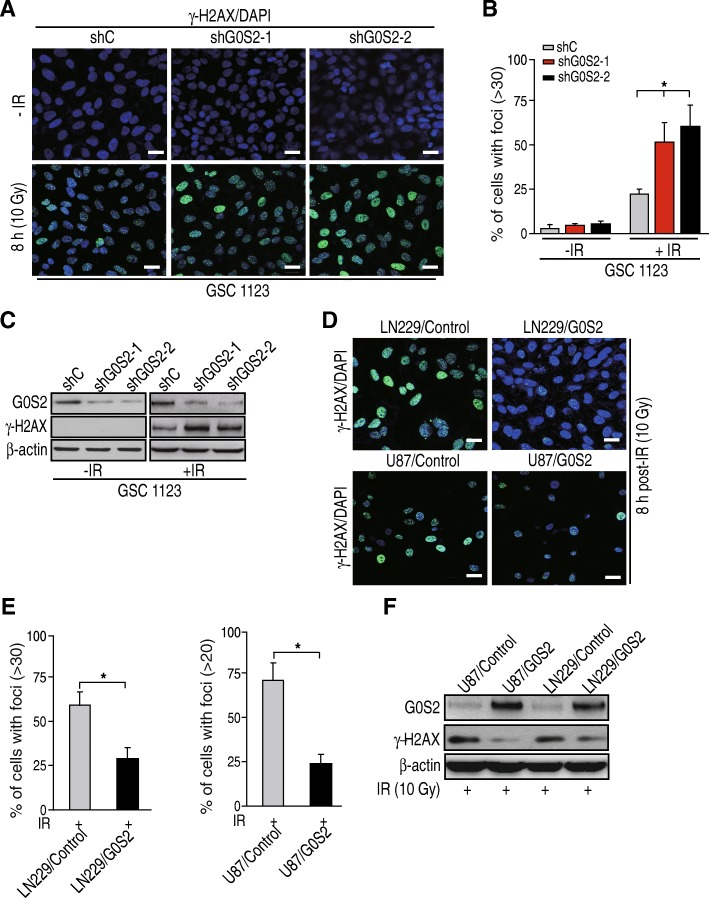
G0S2 regulates glioma cell DNA repair in response to radiation. **a** Representative images of γ-H2AX (p-S139- H2AX) staining of glioma GSC1123/shC, GSC1123/shG0S2–1 and GSC1123/shG0S2–2 cells at 8 h with or without with 10 Gy irradiation. Scale bars: 50 μm. **b** Quantitative γ-H2AX staining assays of A. Error bars, SD. *, *p* < 0.05. **c** WB analyses of effect of G0S2 knockdown on γ-H2AX expression in A. Data represent two independent experiments with similar results. **d** Overexpression of G0S2 inhibited DNA damage induced by radiation. Representative images of γ-H2AX staining of glioma LN229/shC, LN229/G0S2, U87/shC and U87/G0S2 cells at 8 h post-IR with 10 Gy. Scale bars: 50 μm. **e** Quantitative γ-H2AX staining assays of F. Error bars, SD. *, *p* < 0.05. **f** WB analysis of effects of G0S2-overexpression on γ-H2AX expression in D

Emerging evidence suggests that activation of DNA repair is an important factor for glioma radiation resistance [4, 5]. We hypothesize that G0S2 also regulates glioma radiation response through activation of DNA repair. Since γ-H2AX foci have been widely used as a sensitive indicator for DNA repair [28, 29], we used immunofluorescence staining to test and quantify γ-H2AX foci-positive cells in response to IR in GSC 1123/shC, GSC 1123/shG0S2–1 and 1123/shG0S2–2 cells. As shown in Fig. 4a and b, after 8 h post-IR with 10 Gy, GSC 1123/shC cells showed γ-H2AX foci formation. Compared with the controls, knockdown of G0S2 in GSC 1123 cells significantly enhanced IR-induced γ-H2AX foci-formation (Fig. 4a and b). IR-induced upregulation of γ-H2AX was further confirmed by western blotting assay (Fig. 4c). In contrast, compared with the controls, overexpression of G0S2 in glioma LN229 and U87 glioma cells significantly prevented IR-induced γ-H2AX foci formation in these cells (Fig. 4d and e). Additionally, overexpression of G0S2 also inhibited IR-induced γ-H2AX expression (Fig. 4f). This data suggests that G0S2 involves glioma response to IR treatment through regulating DNA repair pathways.

### G0S2 promotes 53BP1 stability in glioma cells in response to IR

**Fig. 5:**
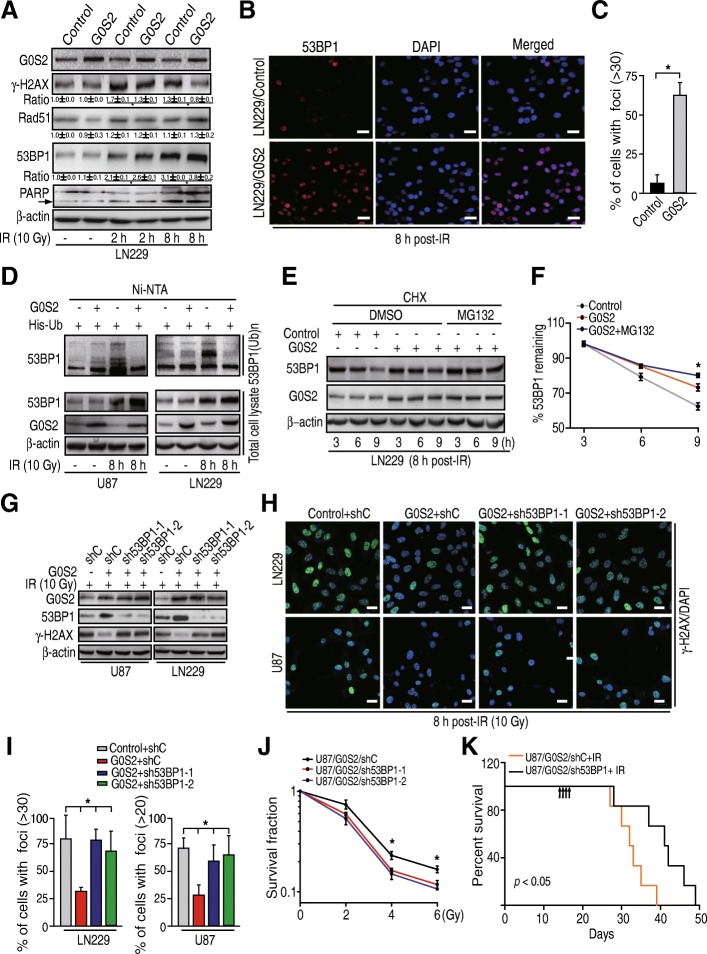
G0S2 promotes 53BP1 stability in glioma cells in response to radiation. **a** WB analyses of effect of G0S2 overexpression on γ-H2AX, Rad51, 53BP1 and PARP expression in response to IR. Arrow, cleaved PARP. **b** Representative images of 53BP1 staining of glioma LN229 with or without overexpression of G0S2 at 8 h post-IR with 10 Gy. Scale bars: 50 μm. **c** Quantitative 53BP1 staining of B. Error bars, SD. *, *p* < 0.05. **d** G0S2 increased 53BP1 ubiquitination in response to IR. His-Ub was transiently transfected into glioma U87 and LN229 cells with or without overexpression of G0S2. Proteins were pulled down with Ni-NTA beads. 53BP1(Ub)n, polyubiquitinated 53BP1. **e** The half-life of 53BP1 in LN229 cells with overexpression of G0S2 was prolonged compared with the control. After 8-h post-IR, cells were treated with CHX (10 μg/ml) for indicated times with or without the proteasome inhibitor MG132 (10 μM). **f** Quantitative 53BP1 protein levels of panel **e** using image **j** software. Error bars, SD. *, *p* < 0.05. **g** Depletion of 53BP1 with two different shRNAs (sh53BP1–1 and sh53BP1–2) increased G0S2-attenuated γ-H2AX expression in response to radiation in glioma LN229 and U87 cells. **h** Representative images of γ-H2AX staining of **g**. Scale bars: 50 μm. **i** Quantitative γ-H2AX staining of **h**. Error bars, SD. *, *p* < 0.05. **j** Clonogenic survival assay of U87/G0S2 cells transduced with control shRNA (shC) or 53BP1 shRNAs (sh53BP1–1 and sh53BP1–2). Colonies formed by surviving cells 26 days after IR are shown. Error bars, SD. *, *p* < 0.05. **k** Survival curves for mice implantated with 5 × 10^5^ cells of U87/G0S2/shC or U87 /G0S2/sh53BP1 and left untreated or given daily dosed 2.5 Gy IR from day 14 to 17 after cell implantation. Arrows, IR treatment times. Data represent two independent experiments with 6 mice per group with similar results. Data in (**a** to **j**) represent two to three independent experiments with similar results

Accumulated evidence has established critical roles for the tumor suppressor p53-binding protein 1 (53BP1) in non-homologous end-joining (NHEJ) double-stand break (DSB) DNA repair [30–32], and Rad51 in homologous recombination (HR) DSB DNA repair [33]. In addition, 53BP1 and Rad51 are important for glioma tumorigenesis [6, 34]. Based on our results above, we hypothesized that G0S2 enhances radiation resistance of gliomas through regulation of Rad51 or 53BP1. As shown in Fig. 5a, LN229/Control and LN229/G0S2 cells without IR treatment showed basal levels of γ-H2AX, Rad51 and 53BP1 protein expression. At 2 h and 8 h post-IR, protein levels of γ-H2AX, Rad51 and 53BP1 were elevated in both cell lines. Compared with LN229/Control cells, the levels of γ-H2AX protein were significantly lower in LN229/G0S2 cells at 2 h and 8 h post-IR whereas the levels of 53BP1 protein were markedly higher (Fig. 5a). The levels of Rad51 protein did not show any differences in LN229/G0S2 cells compared with LN229/Control cells at 2 h and 8 h post-IR treatments, respectively (Fig. 5a). Immunofluorescent staining also indicated that the ratio of 53BP1 foci positive cells were higher in LN229/G0S2 cells compared with LN229/Control cells at 8 h post-IR (Fig. 5b and c). We also determined cell apoptosis in these isogenic LN229 cells in response to IR. As shown in Fig. 5a, levels of cleaved PARP, a marker of cell apoptosis [35] were markedly increased in response to IR. However, levels of cleaved PARP were not affected by overexpression of G0S2 compared with the controls. Lastly, we assessed the expression of *53BP1* mRNA in LN229/G0S2 cells compared with in LN229/Control cells in response to IR. However, we did not find appreciable changes of the levels of *53BP1* mRNA in these isogenic cells (Additional file 2: Figure S2), suggesting that alterations are regulated at the post-translational level. In sum, these results show that 53BP1 protein expression is regulated by G0S2 in glioma cell responses to IR treatment.

Since 53BP1 stability is regulated by ubiquitination in DNA repair [36, 37], we tested if G0S2 regulates 53BP1 ubiquitination and stability in glioma cells in response to IR. As shown in Fig. 5d, comparing untreated with irradiated glioma cells, 53BP1 unbiquitination was significantly enhanced by IR in control U87 and LN229 cells at 8-h post-IR. However, G0S2 overexpression inhibited IR-stimulated 53BP1 ubiquitination compared with the controls. We subsequently assessed the protein stability of 53BP1 in the presence of cycloheximide (CHX) that blocks de novo protein synthesis with or without G0S2 overexpression in response to IR. As shown in Fig. 5e and f, overexpression of G0S2 markedly inhibited 53BP1 protein degradation compared with the control. Moreover, the treatment with MG132, a proteasome inhibitor, significantly delayed 53BP1 degradation in G0S2-overexpressed cells. This data supports the notion that G0S2 regulates ubiquitination and proteasome-dependent degradation of 53BP1 in gliomas in response to IR.

To further demonstrate that G0S2 regulates glioma radioresistance through 53BP1, we knocked down 53BP1 using shRNAs in U87/G0S2 and LN229/G0S2 cells and determined γ-H2AX expression and γ-H2AX foci formation in response to IR. As shown in Fig. 5g, knockdown of 53BP1 with two different shRNAs, sh53BP1–1 and sh53BP1–2, increased γ-H2AX protein levels in U87/G0S2 and LN229/G0S2 cells compared with the controls in response to IR. Depletion of 53BP1 markedly attenuated γ-H2AX foci cell formation in U87/G0S2 and LN229/G0S2 cells compared with the controls (Fig. 5h and i). Moreover, knockdown of 53BP1 enhanced U87/G0S2 cell radiosensitivity compared with the control (Fig. 5j), and significantly extended animal survival with median survival times of 38.5 days compared to 32.5 days of control animals (Fig. 5k). Taken together, these data support that G0S2 regulates glioma radioresistance through mediating 53BP1 stability in response to IR.

### G0S2 activates mTOR-S6K signaling and thereby inhibits RNF168 expression and RNF168-mediated 53BP1 ubiquitination in response to IR

**Fig. 6:**
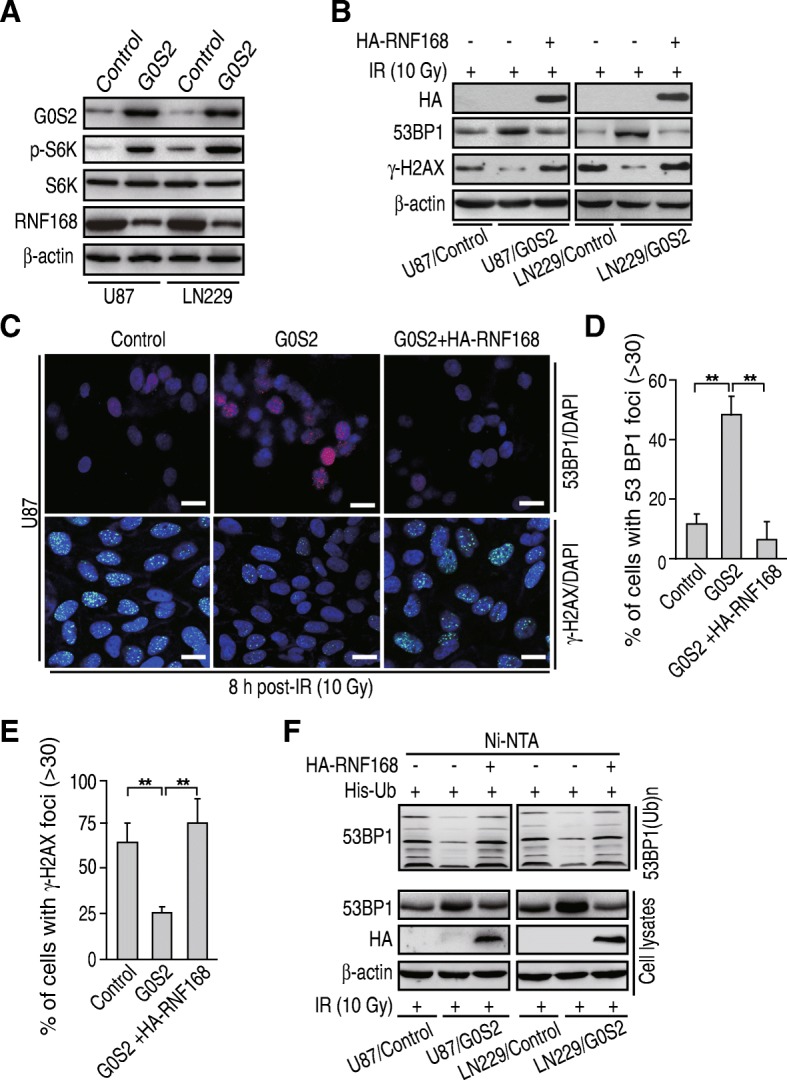
G0S2 activates mTOR-S6K signaling and thereby inhibits RNF168 expression and RNF168-mediated 53BP1 ubiquitination in response to IR. **a** WB analysis of p-S6K and RNF168 in U87 and LN229 cells with or without G0S2 overexpression. β-actin and S6K were used as controls. **b** Ectopic expression of RNF168 decreased 53BP1 protein expression upregulated by G0S2 overexpression at 8 h post-IR with 10 Gy, whereas it increased γ-H2AX expression inhibited by G0S2 overexpression. HA-RNF168 cDNA was transiently transfected into U87/G0S2 and LN229/G0S2 cells. **c** Representative images of 53BP1 staining at 8 h post-IR with 10 Gy. Cells were from **b**. Scale bars: 50 μm. **d**-**e**. Quantitative 53BP1 foci cells (**d**) and γ-H2AX foci cells (**e**) of **c**. Error bars, SD. *, *p* < 0.05. **f** RNF168 overexpression increased 53BP1 ubiquitination inhibited by G0S2 overexpression in response to IR. His-Ub and HA-RNF168 were transiently transfected into glioma U87/G0S2 and LN229/G0S2 cells. Data in (**a**, **b** and **f**) represent two to three independent experiments with similar results

E3 ubiquitin ligase RNF168 mediates 53BP1 stability and sensitivity of cancer cells to DNA damaging agents and irradiation [37, 38], and RNF168 expression is inhibited by rapamycin (mTOR)-ribosomal S6 kinase (S6K) signaling [38]. Moreover, exogenously supplied oleic acid (OA) activates mTOR-S6K signaling [39]. We hypothesized that G0S2 reduces lipid droplet turnover and thereby activates the mechanistic target of rapamycin (mTOR)-ribosomal S6 kinase (S6K) signaling, reduces E3 ligase RNF168 expression, inhibits RNF168-mediated protein ubiquitination of 53BP1, and increases 53BP1 stability in response to IR. To assess this, we detected effects of G0S2 overexpression on S6K phosphorylation (p-S6K) and RNF168 protein expression in U87 and LN229 cells. As shown in Fig. 6a, overexpression of G0S2 significantly increased p-S6K levels and decreased RNF168 protein expression. We then transiently transfected RNF168 into U87/G0S2 and LN229/G0S2 cells, and found that ectopic expression of RNF168 inhibited 53BP1 expression (Fig. 6b) and 53BP foci cell formation (Fig. 6c and d) upregulated by G0S2 overexpression in indicated glioma cells compared to the controls at 8 h post IR, whereas it increased γ-H2AX expression (Fig. 6b) and γ-H2AX foci cell formation inhibited by G0S2 overexpression (Fig. 6c and e). We further found that overexpression of RNF168 enhanced 53BP1 ubiquitination inhibited by G0S2 overexpression in U87 and LN229 cells in response to IR (Fig. 6f). Our results demonstrate that G0S2 regulates glioma radioresistance through mTOR/S6K/ RNF168/53BP1-regulated DNA repair.

## Discussion

In this study, we described a novel mechanism of radiation resistance in GBM linked to upregulation of G0S2. G0S2 is upregulated in radioresistant GSCs and elevated in clinical GBM biopsies. Modulation of G0S2 expression affects GBM responses to IR treatments in vitro and in vivo through mTOR/S6K/RNF168/53BP1-regulated DNA repair, suggesting G0S2 as a potential mediator of glioma responses to IR. Moreover, G0S2-induced radioresistance is related with G0S2-mediated lipid droplet stability.

Our data demonstrate for the first time that G0S2 functions as a mediator of radiation resistance in gliomas. G0S2 is a 12 kDa small protein that was initially shown to be involved in cell cycle progression [7, 8]. G0S2 was then characterized as an inhibitor of adipose triglyceride lipase (ATGL) to regulate lipolysis [12]. Recently, G0S2 was described as a positive regulator of hypoxia-induced ATP production [9]. G0S2 was rapidly and transiently induced by hypoxia, and physiological enhancement of G0S2 expression prevented cells from ATP depletion and induced a cellular tolerance for hypoxia stress [9]. G0S2 also was identified as a NF-κB-dependent downstream factor of TNF-α in primary foreskin fibroblasts [10]. PN-MES transition of GSCs promoted radioresistance in a TNF-α/NF-kB-dependent manner [40]. Here, we show that G0S2 modulates radiation responses of gliomas. Expression level of G0S2 was upregulated in radioresistant GSCs. Knockdown of G0S2 by shRNAs sensitized glioma cells to IR treatments in vitro and glioma tumorigenicity. In contrast, overexpression of G0S2 increased the resistance of glioma cell and tumor to IR treatments, demonstrating an undescribed function of G0S2 in glioma radioresistance.

Our results suggest that G0S2 mediates glioma radioresistance through 53BP1-regulated DNA repair. Accumulated data demonstrate that 53BP1 is critical in radiation response of tumors [36, 37], including glioma [6]. Depletion of endogenous 53BP1 sensitized glioma cells to IR treatment in vitro and in vivo [6]*.* Moreover, 53BP1 stability was shown to be regulated by ubiquitination in response to irradiation [31, 37]. Here, our results support this notion and show that G0S2 overexpression inhibited 53BP1 ubiquitination and upregulated 53BP1 foci cell formation in glioma cells, and thereby reduced γ-H2AX expression and γ-H2AX foci cell formation in response to irradiation. Consistent with this, depletion of 53BP1 inhibited G0S2 overexpression-induced radioresistance.

Our results also suggest that G0S2 mediates 53BP1 ubiquitination and stability through mTOR/S6K signaling-regulated RNF168 expression. 53BP1 stability is regulated by E3 ubiquitin ligase RNF168 in DNA damage [37, 38]. mTOR-S6K phosphorylates RNF168 at Ser60 and thereby inhibits its E3 ligase activity, accelerates its proteolysis, and impairs its function in DNA damage response [38]. Additionally, mTOR-S6K signaling is activated by exogenously supplied oleic acid (OA), a monounsaturated omega-9 fatty acid [39]. In this study, we show that lipolytic inhibitor G0S2 reduces lipid droplet turnover and thereby activates mTOR-S6K signaling, inhibits E3 ligase RNF168 expression and RNF168- mediated 53BP1 protein ubiquitination in response to IR. Thus, our data suggest that G0S2 regulates 53BP1 stability and DNA repair through mTOR-S6K signaling-mediated RNF168.

Previous studies have shown G0S2 to be both oncogenic and tumor suppressive. *G0S2* was found epigenetically silenced in lung cancer and breast cancer lines [10], and engineered expression of G0S2 induced cell apoptosis through G0S2 binding to and antagonizing Bcl-2 in a lung and a colon cancer cell line [10] but not in breast cancer cell lines [41]. In a chronic myeloid leukemia cell line, K562, *G0S2* gene was found to be silenced by gene methylation, and upregulation of G0S2 expression by retroviral transduction or treatment with 5-azacytidine inhibited the proliferation of K562 cells both in vitro and in a xenograft model [42]. Although Zagani et al. [43] demonstrated that Eμ-Myc transgenic mouse model was not the correct model to conduct studies on G0S2, they found deletion of the *G0S2* gene in mice did not show any effect on the latency of cancer progression in the Eμ-Myc model of lymphoma [43]. By analyzing The Cancer Genome Atlas (TCGA), Fukunaga et al. showed that pateients in the higher G0S2 expression group had a poorer prognosis [18]. They also demonstrated that G0S2 expression levels were higher in recurrent tumor specimens that that at the initial diagnosis in the same patients [18]. Here in this study, we show that G0S2 promotes tumor growth in gliomas. Compared with paired normal brain tissues, we further found that the level of G0S2 expression was elevated in clinical tumor specimens. Patients with high level of G0S2 expression in GBM have a poorer prognosis compared with those with low level of G0S2 expression. Our analysis of GSE7969 and GDS1962 datasets supports this observation demonstrating that G0S2 expression was increased in GBM tumors compared with normal brain tissues. By using genetic approaches targeting G0S2, we found that shRNA knockdown of G0S2 inhibited glioma tumorigenesis in vivo. Conversely, overexpression of G0S2 promoted tumor growth in orthotopic glioma models. The differences between our results and some of earlier studies of G0S2 in other cancers could reflect context-dependent mechanisms of action in different type of cancers.

That G0S2 functions as an oncogene or tumor suppressor may be related with G0S2 lipolytic inhibitor activity. G0S2 suppressed mouse embryonic fibroblasts (MEF) oncogenic transformation induced by overexpression of HRAS or EGFR, which was independent of G0S2 lipolytic inhibitor function [41]. Lipid droplets were shown to be inversely correlated with GBM patient survival [14]. Depletion of SOAT1, an inhibitor of lipid droplet formation, suppressed GBM growth [14]. In this study, we demonstrate that G0S2-induced radioresistance is related with G0S2-regulated lipid droplet stability in gliomas. Knockdown of G0S2 promoted lipid droplet turnover, cell apoptosis, inhibited radioresistance in GSCs, and extended xenograft tumor animal survival. Consistent with this, oleic acid treatment promoted lipid droplet formation and reduced G0S2 shRNA-inhibited cell apoptosis induced by IR. The molecular insight of relation between lipid droplet turnover and tumorigenicity warrants further inverstigation.

## Conclusions

In summary, our results demonstrate a previously unknown function of G0S2 in enhancing glioma radioresistance through regulation of 53BP1 protein stability, which is related with G0S2 lipolytic inhibitor function. The newly elucidated roles of G0S2 in glioma radioresistance also provide a strong rationale for targeting this molecule in clinical treatment of human gliomas.

## Additional files

Additional file 1: **Figure S1.** Establish redioresistant glioma stem cells. A. Experiment protocol for establishment of radioresistant GSCs. B. Flow cytometric analysis for GSC 1123-C and GSC 1123-R cells stained with annexin V and propidium iodide at indicated time points after exposure to 6 Gy of ionization radiation or not. C. Quantification of apoptotic cells from B. Error bars SD. *, *p* < 0.05. D. Representative images of clonogenic survival assay of GSC 1123-C and GSC 1123-R cells. Colonies formed by surviving cells 15 days after IR are shown. Scale bars. 1 mm. E. Quantification of colonies in D. Error bars, SD. *, *p* < 0.05. (EPS 6598 kb)

Additional file 2: **Figure S2.** Quantitative real-time-PCR (QRT-PCR) analysis of *53BP1* mRNA expression in LN229/G0S2 and LN229/Control cells after IR treatment. *ACTB* was used as an internal control. Errors bars, SD. *, *p* < 0.05. (EPS 837 kb)

## Abbreviations

*ALDH1A3*: Aldehyde dehydrogenase 1A3
G0S2: G0/G1 switch gene 2
GBM: Glioblastoma
GSC: Glioma stem-like cell
IR: Ionizing radiation
MES: Mesenchymal
mTOR: mammalian target of rapamycin
NSC: Neural stem cell
OA: Oleic acid
PN: Proneural
S6K: Ribosomal S6 kinase

## References

[1] Stupp R, Mason WP, van den Bent MJ, Weller M, Fisher B, Taphoorn MJ (2005). Radiotherapy plus concomitant and adjuvant temozolomide for glioblastoma. N Engl J Med.

[2] Chinot OL, Wick W, Mason W, Henriksson R, Saran F, Nishikawa R (2014). Bevacizumab plus radiotherapy-temozolomide for newly diagnosed glioblastoma. N Engl J Med.

[3] Moding EJ, Kastan MB, Kirsch DG (2013). Strategies for optimizing the response of cancer and normal tissues to radiation. Nat Rev Drug Discov.

[4] Bao S, Wu Q, McLendon RE, Hao Y, Shi Q, Hjelmeland AB (2006). Glioma stem cells promote radioresistance by preferential activation of the DNA damage response. Nature..

[5] Brett-Morris A, Wright BM, Seo Y, Pasupuleti V, Zhang J, Lu J (2014). The polyamine catabolic enzyme SAT1 modulates tumorigenesis and radiation response in GBM. Cancer Res.

[6] Squatrito M, Vanoli F, Schultz N, Jasin M, Holland EC (2012). 53BP1 is a haploinsufficient tumor suppressor and protects cells from radiation response in glioma. Cancer Res.

[7] Russell L, Forsdyke DR (1991). A human putative lymphocyte G0/G1 switch gene containing a CpG-rich island encodes a small basic protein with the potential to be phosphorylated. DNA Cell Biol.

[8] Siderovski DP, Blum S, Forsdyke RE, Forsdyke DR (1990). A set of human putative lymphocyte G0/G1 switch genes includes genes homologous to rodent cytokine and zinc finger protein-encoding genes. DNA Cell Biol.

[9] Kioka H, Kato H, Fujikawa M, Tsukamoto O, Suzuki T, Imamura H (2014). Evaluation of intramitochondrial ATP levels identifies G0/G1 switch gene 2 as a positive regulator of oxidative phosphorylation. Proc Natl Acad Sci U S A.

[10] Welch C, Santra MK, El-Assaad W, Zhu X, Huber WE, Keys RA (2009). Identification of a protein, G0S2, that lacks Bcl-2 homology domains and interacts with and antagonizes Bcl-2. Cancer Res.

[11] Zandbergen F, Mandard S, Escher P, Tan NS, Patsouris D, Jatkoe T (2005). The G0/G1 switch gene 2 is a novel PPAR target gene. Biochem J.

[12] Yang X, Lu X, Lombes M, Rha GB, Chi YI, Guerin TM (2010). The G(0)/G(1) switch gene 2 regulates adipose lipolysis through association with adipose triglyceride lipase. Cell Metab.

[13] Yamada T, Park CS, Burns A, Nakada D, Lacorazza HD (2012). The cytosolic protein G0S2 maintains quiescence in hematopoietic stem cells. PLoS One.

[14] Geng F, Cheng X, Wu X, Yoo JY, Cheng C, Guo JY (2016). Inhibition of SOAT1 suppresses glioblastoma growth via blocking SREBP-1-mediated lipogenesis. Clin Cancer Res.

[15] Zagani R, El-Assaad W, Gamache I, Teodoro JG (2015). Inhibition of adipose triglyceride lipase (ATGL) by the putative tumor suppressor G0S2 or a small molecule inhibitor attenuates the growth of cancer cells. Oncotarget..

[16] Kusakabe M, Kutomi T, Watanabe K, Emoto N, Aki N, Kage H (2010). Identification of G0S2 as a gene frequently methylated in squamous lung cancer by combination of in silico and experimental approaches. Int J Cancer.

[17] Kusakabe M, Watanabe K, Emoto N, Aki N, Kage H, Nagase T (2009). Impact of DNA demethylation of the G0S2 gene on the transcription of G0S2 in squamous lung cancer cell lines with or without nuclear receptor agonists. Biochem Biophys Res Commun.

[18] Fukunaga T, Fujita Y, Kishima H, Yamashita T (2018). Methylation dependent down-regulation of G0S2 leads to suppression of invasion and improved prognosis of IDH1-mutant glioma. PLoS One.

[19] Mao P, Joshi K, Li J, Kim SH, Li P, Santana-Santos L (2013). Mesenchymal glioma stem cells are maintained by activated glycolytic metabolism involving aldehyde dehydrogenase 1A3. Proc Natl Acad Sci U S A.

[20] Feng H, Lopez GY, Kim CK, Alvarez A, Duncan CG, Nishikawa R (2014). EGFR phosphorylation of DCBLD2 recruits TRAF6 and stimulates AKT-promoted tumorigenesis. J Clin Invest.

[21] Zhang L, Zhang W, Li Y, Alvarez A, Li Z, Wang Y (2016). SHP-2-upregulated ZEB1 is important for PDGFRalpha-driven glioma epithelial-mesenchymal transition and invasion in mice and humans. Oncogene..

[22] Franken NA, Rodermond HM, Stap J, Haveman J, van Bree C (2006). Clonogenic assay of cells in vitro. Nat Protoc.

[23] Liu KW, Feng H, Bachoo R, Kazlauskas A, Smith EM, Symes K (2011). SHP-2/PTPN11 mediates gliomagenesis driven by PDGFRA and INK4A/ARF aberrations in mice and humans. J Clin Invest.

[24] Murat A, Migliavacca E, Gorlia T, Lambiv WL, Shay T, Hamou MF (2008). Stem cell-related “self-renewal” signature and high epidermal growth factor receptor expression associated with resistance to concomitant chemoradiotherapy in glioblastoma. J Clin Oncol.

[25] Sun L, Hui AM, Su Q, Vortmeyer A, Kotliarov Y, Pastorino S (2006). Neuronal and glioma-derived stem cell factor induces angiogenesis within the brain. Cancer Cell.

[26] Lee Y, Scheck AC, Cloughesy TF, Lai A, Dong J, Farooqi HK (2008). Gene expression analysis of glioblastomas identifies the major molecular basis for the prognostic benefit of younger age. BMC Med Genet.

[27] Qiu B, Simon MC (2016). BODIPY 493/503 Staining of Neutral Lipid Droplets for Microscopy and Quantification by Flow Cytometry. Bio Protoc.

[28] Dickey JS, Redon CE, Nakamura AJ, Baird BJ, Sedelnikova OA, Bonner WM (2009). H2AX: functional roles and potential applications. Chromosoma..

[29] Willers H, Gheorghiu L, Liu Q, Efstathiou JA, Wirth LJ, Krause M (2015). DNA damage response assessments in human tumor samples provide functional biomarkers of Radiosensitivity. Semin Radiat Oncol.

[30] Zimmermann M, Lottersberger F, Buonomo SB, Sfeir A, de Lange T (2013). 53BP1 regulates DSB repair using Rif1 to control 5′ end resection. Science..

[31] Chapman JR, Barral P, Vannier JB, Borel V, Steger M, Tomas-Loba A (2013). RIF1 is essential for 53BP1-dependent nonhomologous end joining and suppression of DNA double-strand break resection. Mol Cell.

[32] Di Virgilio M, Callen E, Yamane A, Zhang W, Jankovic M, Gitlin AD (2013). Rif1 prevents resection of DNA breaks and promotes immunoglobulin class switching. Science..

[33] Arias-Lopez C, Lazaro-Trueba I, Kerr P, Lord CJ, Dexter T, Iravani M (2006). p53 modulates homologous recombination by transcriptional regulation of the RAD51 gene. EMBO Rep.

[34] Westermark UK, Lindberg N, Roswall P, Brasater D, Helgadottir HR, Hede SM (2011). RAD51 can inhibit PDGF-B-induced gliomagenesis and genomic instability. Neuro-Oncology.

[35] Boulares AH, Yakovlev AG, Ivanova V, Stoica BA, Wang G, Iyer S (1999). Role of poly(ADP-ribose) polymerase (PARP) cleavage in apoptosis. Caspase 3-resistant PARP mutant increases rates of apoptosis in transfected cells. J Biol Chem.

[36] Han X, Zhang L, Chung J, Mayca Pozo F, Tran A, Seachrist DD (2014). UbcH7 regulates 53BP1 stability and DSB repair. Proc Natl Acad Sci U S A.

[37] Hu Y, Wang C, Huang K, Xia F, Parvin JD, Mondal N (2014). Regulation of 53BP1 protein stability by RNF8 and RNF168 is important for efficient DNA double-strand break repair. PLoS One.

[38] Xie X, Hu H, Tong X, Li L, Liu X, Chen M (2018). The mTOR-S6K pathway links growth signalling to DNA damage response by targeting RNF168. Nat Cell Biol.

[39] Menon D, Salloum D, Bernfeld E, Gorodetsky E, Akselrod A, Frias MA (2017). Lipid sensing by mTOR complexes via de novo synthesis of phosphatidic acid. J Biol Chem.

[40] Bhat KP, Balasubramaniyan V, Vaillant B, Ezhilarasan R, Hummelink K, Hollingsworth F (2013). Mesenchymal differentiation mediated by NF-kappaB promotes radiation resistance in glioblastoma. Cancer Cell.

[41] Yim CY, Sekula DJ, Hever-Jardine MP, Liu X, Warzecha JM, Tam J (2016). G0S2 suppresses oncogenic transformation by repressing a MYC-regulated transcriptional program. Cancer Res.

[42] Yamada T, Park CS, Shen Y, Rabin KR, Lacorazza HD (2014). G0S2 inhibits the proliferation of K562 cells by interacting with nucleolin in the cytosol. Leuk Res.

[43] Zagani R, Gamache I, Teodoro JG (2016). Deletion of the putative tumor suppressor gene, G0s2, does not affect progression of emu-Myc driven lymphomas in mice. Leuk Res.

[44] Phillips HS, Kharbanda S, Chen R, Forrest WF, Soriano RH, Wu TD (2006). Molecular subclasses of high-grade glioma predict prognosis, delineate a pattern of disease progression, and resemble stages in neurogenesis. Cancer Cell.

