# Correction to: Lipolytic inhibitor G0S2 modulates glioma stem-like cell radiation response

**DOI:** 10.1186/s13046-019-1335-4

**Published:** 2019-07-29

**Authors:** Yinfang Wang, Yanli Hou, Weiwei Zhang, Angel A. Alvarez, Yongrui Bai, Bo Hu, Shi-Yuan Cheng, Kun Yang, Yanxin Li, Haizhong Feng

**Affiliations:** 1 State Key Laboratory of Oncogenes and Related Genes, Renji-Med X Clinical Stem Cell Research Center, Ren Ji Hospital, Shanghai Cancer Institute, School of Medicine, Shanghai Jiao Tong University, Shanghai, 200127 China; 2 Department of Radiotherapy, Ren Ji Hospital, School of Medicine, Shanghai Jiao Tong University, Shanghai, 200127 China; 3 Ken and Ruth Davee Department of Neurology, Lou & Jean Malnati Brain Tumor Institute, The Robert H. Lurie Comprehensive Cancer Center, Northwestern University Feinberg School of Medicine, Chicago, IL 60611 USA; 4 Department of Neurosurgery, The First Affiliated Hospital of Hainan Medical University, Haikou, 570102 Hainan China; 5 Key Laboratory of Pediatric Hematology and Oncology Ministry of Health, Pediatric Translational Medicine Institute, Shanghai Children’s Medical Center, School of Medicine, Shanghai Jiao Tong University, Shanghai, 200127 China

**Correction to: J Exp Clin Cancer Res**

**https://doi.org/10.1186/s13046-019-1151-x**

In the original publication of this article [1], the Gene Expression Omnibus (GEO) accession code GSE79772 is wrong. The correct accession code should be GSE86213.
